# Mendelian randomization reveals association between retinal thickness and non-motor symptoms of Parkinson’s disease

**DOI:** 10.1038/s41531-023-00611-z

**Published:** 2023-12-13

**Authors:** Hang Zhou, Bibiao Shen, Zifeng Huang, Shuzhen Zhu, Wanlin Yang, Fen Xie, Yuqi Luo, Feilan Yuan, Zhaohua Zhu, Chao Deng, Wenhua Zheng, Chengwu Yang, Chin-Hsien Lin, Bin Xiao, Eng-King Tan, Qing Wang

**Affiliations:** 1 Department of Neurology, Zhujiang Hospital of Southern Medical University, Guangzhou, Guangdong 510282 P.R. China; 2 Clinical Research Centre, Orthopedic Centre, Zhujiang Hospital of Southern Medical University, Guangzhou, Guangdong 510282 P.R. China; 3 School of Medical, Indigenous and Health Sciences, and Molecular Horizons, University of Wollongong, Wollongong, Australia; 4 Centre of Reproduction, Development & Aging and Institute of Translation Medicine, Faculty of Health Sciences, University of Macau, Avenida de Universidade, Taipa, Macau China; 5 Division of Biostatistics and Health Services Research, Department of Population and Quantitative Health Sciences, T.H. Chan School of Medicine, UMass Chan Medical School, Massachusetts, 01605 USA; 6 Department of Neurology, National Taiwan University Hospital, Taipei, Taiwan; 7 Department of Neurology, National Neuroscience Institute, Singapore General Hospital, Singapore; Duke-NUS Medical School, Singapore, Singapore

**Keywords:** Neurodegeneration, Parkinson's disease, Epidemiology

## Abstract

Retinal thickness is related to Parkinson’s disease (PD), but its association with the severity of PD is still unclear. We conducted a Mendelian randomized (MR) study to explore the association between retinal thickness and PD. For the two-sample MR analysis, the summary statistics obtained from genome-wide association studies on the thickness of Retinal nerve fiber layer (RNFL) and ganglion cell inner plexiform layer (GCIPL) were employed as exposure, while the summary statistics associated with PD were used as the outcome. The primary approach utilized was inverse variance weighted. To correct for multiple testing, the false discovery rate (FDR) was employed. For sensitivity analysis, an array of robust MR methods was utilized. We found genetically predicted significant association between reduced RNFL thickness and a reduced risk of constipation in PD (odds ratio [OR] = 0.854, 95% confidence interval [CI] (0.782, 0.933), *P* < 0.001, FDR-corrected *P* = 0.018). Genetically predicted reduced RNFL thickness was associated with a reduced Unified Parkinson’s Disease Rating Scale total score (*β* = −0.042, 95% CI (−0.079, 0.005), *P* = 0.025), and reduced GCIPL thickness was associated with a lower risk of constipation (OR = 0.901, 95% CI (0.821, 0.988), *P* = 0.027) but a higher risk of depression (OR = 1.103, 95% CI (1.016, 1.198), *P* = 0.020), insomnia (OR = 1.090, 95% CI (1.013, 1.172), *P* = 0.021), and rapid eye movement sleep behaviour disorder (RBD) (OR = 1.198, 95% CI (1.061, 1.352), *P* = 0.003). In conclusion, we identify an association between retinal thickness and non-motor symptoms (constipation, depression, insomnia and RBD) in PD, highlighting the potential of retinal thickness as a biomarker for PD nonmotor symptoms.

## Introduction

Parkinson’s disease (PD) is a prevalent neurodegenerative disorder^1–4^. Typical motor symptoms of PD include rigidity, bradykinesia, and resting tremors^5–9^. However, before the onset of these motor symptoms, PD patients suffer from non-motor symptoms for several years. These non-motor symptoms significantly impact their quality of life^10,11^. Consequently, there is growing attention on the identification and management of PD-related non-motor symptoms.

Visual impairment represents a prevalent non-motor symptom in PD, primarily characterized by decreased color discrimination and contrast sensitivity^12,13^. Moreover, the severity of visual impairment in patients with PD is also related to the motor impairments^14,15^ as well as other non-motor symptoms such as depression, anxiety, and cognition impairment^15–17^. Visual impairments in PD can be partly attributed to degenerative changes in the retina^18,19^. Some observational studies indicate that the retinas of patients with PD show degenerative alterations characterized by decreased thickness in the Retinal Nerve Fiber Layer (RNFL) and the Ganglion Cell-Inner Plexiform Layer (GCIPL)^20–22^. Such alterations correlate with both motor^23^ and non-motor symptoms of PD^24,25^.

Concerning underlying mechanisms, contemporary perspectives propose that changes in the retina, as an extension of the central nervous system, might mirror pathological changes in the brain^26–28^. Imaging study has shown a correlation between reduced retinal thickness and the loss of nigral dopaminergic neurons^24^. A reduction in specific retinal cells could potentially lead to symptoms related to PD. Melanin-containing retinal ganglion cells (mRGCs), integral to retinal photoreceptors, significantly influence human circadian rhythms and melatonin secretion^29^. Studies suggest that a decrease in these retinal cells correlates with circadian rhythm disorders and sleep disorders in patients with PD, which might further lead to cognitive and emotional disorders^30,31^. Therefore, monitoring retinal thickness might serve as a biomarker for evaluating the risk and progression of PD. However, existing evidence primarily demonstrates associative links, while definitive causal relationships has yet to be established.

Mendelian Randomization (MR) is an epidemiological method that leverages genetic principles to deduce the causal relationship between exposures and outcomes. The foundation of MR rests on Mendel’s laws of genetics, which posit that genetic variants are randomly distributed at conception. This random distribution of genetic variants acts as a natural experiment, providing a framework analogous to the randomization in randomized controlled trials (RCTs). The core principle of MR involves utilizing genetic variants as instrumental proxies for the exposures and outcomes of interest. These genetic variants, established at conception and remaining constant throughout life, are impervious to external environmental factors or behaviors. This intrinsic property of genetic variants ensures that MR analysis minimizes susceptibility to confoundings and reverse causality biases commonly seen in the observational studies^32–35^.

Genome-wide association study (GWAS) studies have identified numerous single nucleotide polymorphism (SNP) sites associated with PD^36–38^ and retinal thickness^39^, suggesting that genetic variations are pivotal in both. Given the challenges in conducting RCT studies addressing retinal structural degeneration, MR has emerged as a pivotal method in investigating the causal relationship between retinal thickness and PD. Therefore,in this study, we explore the potential causal relationship between retinal thickness and PD, focusing on non-motor symptoms, using MR. We posit that a reduction in retinal thickness may be linked to sleep disorders and related non-motor symptoms of PD. Concurrently, we also examined the relationship between retinal thickness and the motor symptoms of PD. This study was conducted following the STROBE-MR^40^ guidelines to ensure transparency (Supplementary Table 6).

## Results

### Characteristics of the chosen SNPs

**Fig. 1:**
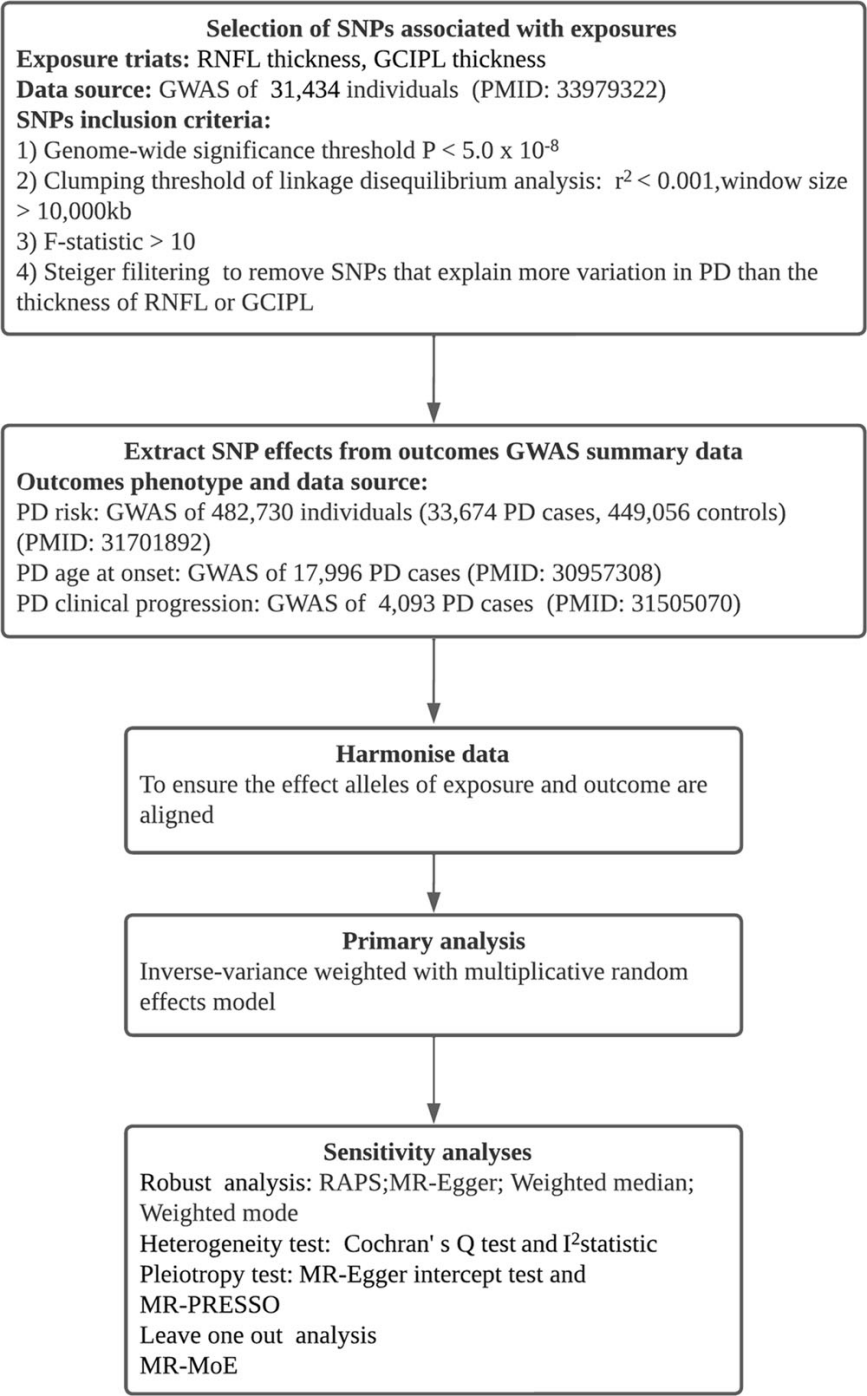
Flow chart of a two-sample Mendelian randomization study of retinal thickness and Parkinson’s disease. The diagram presents our Mendelian randomization analysis approach to examine the association between retinal thickness and outcomes of Parkinson’s disease (PD), utilizing summary data from Genome-Wide Association Studies (GWAS). The procedure entails the selection of specific SNPs based on predefined criteria, extracting pertinent genetic effects, harmonizing the dataset, and performing a primary inverse-variance weighted analysis. To guarantee the robustness of outcomes, several sensitivity analyses are conducted, encompassing pleiotropy and heterogeneity tests, as well as other robust analytical methods. Abbreviations: SNP Single nucleotide polymorphism, RNFL Retinal nerve fiber layer, GCIPL Ganglion cell inner plexiform layer, RAPS Robust Adjusted Profile Score, MR-PRESSO Mendelian Randomization Pleiotropy RESidual Sum and Outlier, MR-MoE Mendelian randomization Mixture of Experts.

Figure 1 shows the process of screening SNPs in our study and Supplementary Table 2 shows the results of the correlation evaluation between the SNPs selected and RNFL or GCIPL thickness. Following screening, the number of SNPs associated with either RNFL or GCIPL selected for further analysis ranges from 7 to 21. The F-statistics and PVE results indicate that these SNPs are strongly associated with exposure factors, thus reducing the likelihood of weak instrumental variable bias. Specifically, all employed F-statistics for SNPs exceed 10, and the average F-statistics for each corresponding set of exposure-outcome SNPs range from 42.78 to 61.48. These SNPs account for a variability proportion in the corresponding exposure factors ranging from 0.95% to 3.59%. The *I*^2^_(GX)_ statistic assesses the potential bias arising from weak instrumental variables in the MR-Egger model. In this study, the *I*^2^_(GX)_ statistic for each exposure-outcome SNP set ranged from 0.5 to 0.93. For the SNP set with an *I*^2^_(GX)_ statistic between 0.6 and 0.9, we subsequently performed a SIMEX-corrected MR-Egger model. However, for the SNP set with an *I*^2^_(GX)_ statistic <0.6, the reference value of its MR-Egger model is not significant.

### Association between genetically predicted RNFL and GCIPL thickness and PD risk

**Fig. 2:**
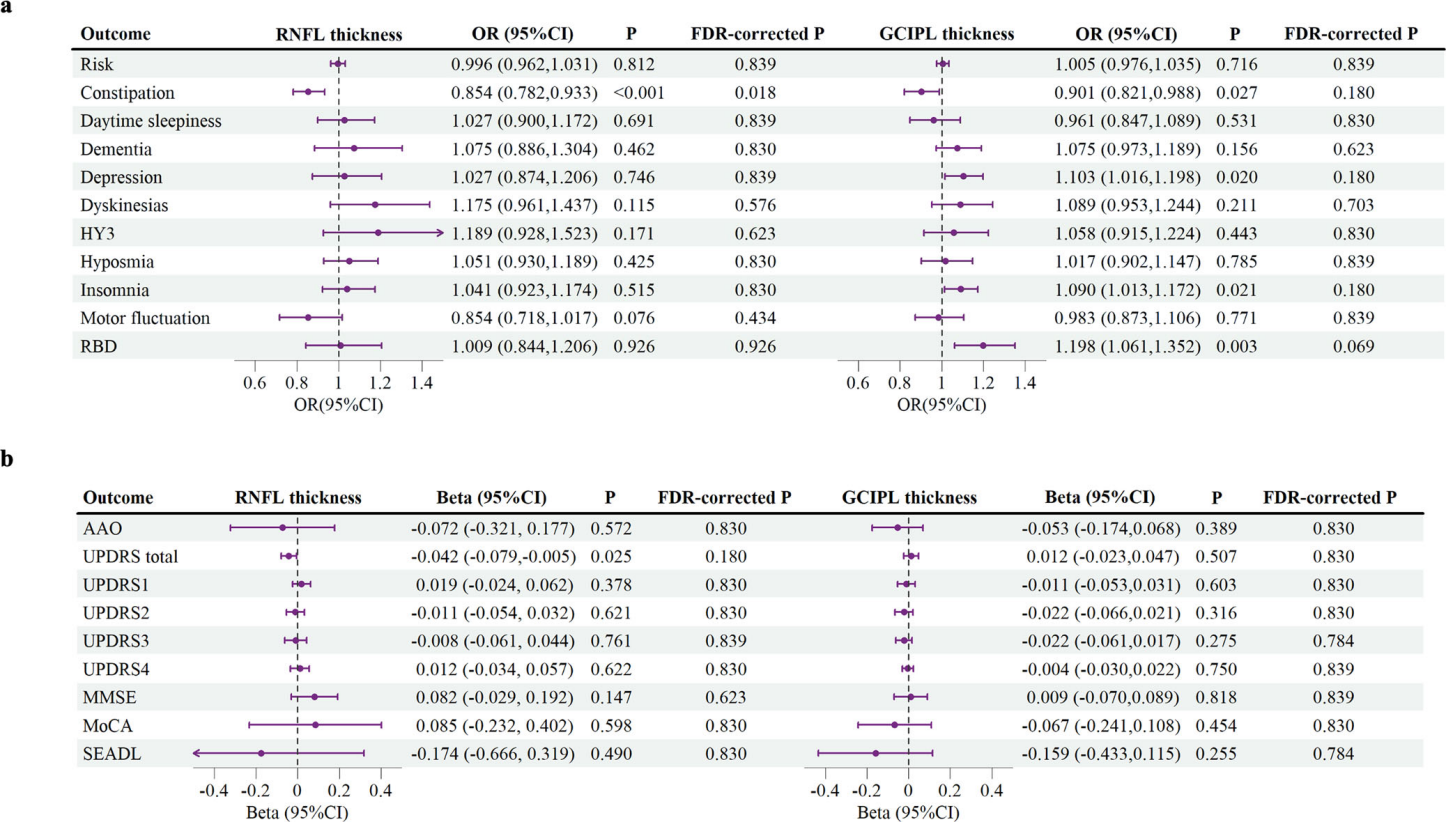
Forest plot of Mendelian randomization results with the inverse-variance weighted method. The inverse-variance weighted Mendelian randomization (MR) estimates of retinal nerve fiber layer (RNFL) thickness or ganglion cell inner plexiform layer (GCIPL) thickness on the (**a**) binary outcomes and (**b**) continuous outcomes of Parkinson’s disease (PD). The forest plot demonstrates a significant association between RNFL thickness and the risk of constipation in PD, with a potential correlation to the Unified Parkinson’s Disease Rating Scale (UPDRS) total score. Furthermore, the data suggest a potential association between GCIPL thickness and the risks of constipation, depression, insomnia, and Rapid eye movement sleep behavior disorder (RBD) in PD. Each circle in the graph represents an inverse-variance weighted estimate for the effect of RNFL or GCIPL thickness on PD. The horizontal line represents the 95% confidence intervals (CI) for the estimates. For binomial outcomes, MR estimates are reported as odds ratios (ORs) along with their corresponding 95% Cls. For continuous outcomes, the MR estimates are reported as betas with their 95% CIs. Abbreviations: FDR False discovery rate, HY3 Hoehn-Yahr stage of 3 or more, AAO Age at Onset, MMSE Mini-Mental State Examination, MoCA Montreal Cognitive Assessment, SEADL Schwab and England Activities of Daily Living Scale.

Figure 2 presents the primary MR analysis results using the IVW model. There was no clear association between genetically predicted RNFL thickness (OR = 0.996, 95% confidence interval [CI] (0.962, 1.031), *P* = 0.812) or GCIPL thickness (OR = 1.005, 95% CI (0.976, 1.035), *P* = 0.716) and PD risk in the present study (Fig. 2a). Likewise, the robust MR model did not establish a correlation between RNFL or GCIPL thickness and PD risk (Tables 1, 2). Regarding the quality control of IVs, there was no evidence of heterogeneity (Supplementary Table 3) or pleiotropy (Supplementary Table 4) in the association analysis between RNFL thickness and PD risk. Although there was significant heterogeneity in the association analysis between GCIPL thickness and PD risk (Cochran’s *Q* = 32.16, *P* = 0.04, *I*^2^ = 38%; Supplementary Table 3), neither the MR-Egger intercept test nor the MR-PRESSO global test detected evident pleiotropy (Supplementary Table 4). This suggests an unlikely presence of directional pleiotropy that might influence effect estimation. Additionally, the robust MR model results in the association analysis between GCIPL thickness and PD risk were consistent with IVW (Table 2), further supporting this perspective. With regard to statistical power, our analysis is sufficiently equipped to detect a 20% alteration in PD risk (Supplementary Table 5).

**Table 1:** Robust Mendelian randomization analysis of association between retinal nerve fiber layer thickness and Parkinson’s disease.

	RAPS		MR-Egger		WME		Weighted MBE	
Outcome	Beta or OR (95%CI)	*P*	Beta or OR (95%CI)	*P*	Beta or OR (95%CI)	*P*	Beta or OR (95%CI)	*P*
Risk	0.996 (0.966,1.027)	0.784	0.941 (0.828,1.069)	0.360	1.006 (0.964,1.051)	0.774	1.018 (0.964,1.075)	0.530
Constipation	0.853 (0.676,1.077)	0.182	1.144 (1.024,1.278)	0.045	0.830 (0.625,1.101)	0.196	0.803 (0.554,1.162)	0.274
Daytime sleepiness	1.027 (0.816,1.293)	0.818	1.126 (0.944,1.343)	0.218	0.994 (0.744,1.328)	0.967	1.018 (0.696,1.489)	0.928
Dementia	1.077 (0.861,1.346)	0.518	0.629 (0.477,0.829)	0.007	1.083 (0.796,1.474)	0.613	1.223 (0.828,1.805)	0.332
Depression	1.027 (0.804,1.312)	0.830	0.564 (0.429,0.741)	0.003	1.010 (0.743,1.373)	0.949	1.009 (0.679,1.499)	0.966
Dyskinesias	1.177 (0.854,1.621)	0.320	0.757 (0.113,5.057)	0.786	1.099 (0.730,1.653)	0.652	1.023 (0.599,1.748)	0.936
HY3	1.191 (0.860,1.651)	0.293	2.098 (1.418,3.105)	0.008	1.245 (0.818,1.895)	0.307	1.345 (0.756,2.395)	0.343
Hyposmia	1.052 (0.839,1.318)	0.662	0.836 (0.643,1.086)	0.212	1.008 (0.751,1.351)	0.960	0.974 (0.648,1.462)	0.900
Insomnia	1.041 (0.872,1.244)	0.656	0.613 (0.504,0.745)	0.000	0.955 (0.758,1.203)	0.694	0.925 (0.668,1.280)	0.645
Motor fluctuation	0.853 (0.675,1.077)	0.182	0.803 (0.197,3.276)	0.770	0.914 (0.682,1.223)	0.543	1.043 (0.644,1.688)	0.869
RBD	1.009 (0.713,1.426)	0.962	1.274 (0.937,1.731)	0.173	1.121 (0.729,1.724)	0.601	1.146 (0.685,1.915)	0.620
AAO	−0.073 (−0.323,0.176)	0.563	0.180 (−0.148,0.509)	0.300	0.017 (−0.346,0.379)	0.928	0.113 (−0.441,0.666)	0.695
UPDRS total	−0.043 (−0.110,0.025)	0.214	−0.058 (−0.126,0.011)	0.133	−0.036 (−0.119,0.048)	0.401	0.002 (−0.110,0.115)	0.969
UPDRS1	0.019 (−0.061, 0.100)	0.637	0.138 (0.041,0.235)	0.019	0.043 (−0.060,0.145)	0.415	0.051 (−0.066,0.167)	0.413
UPDRS2	−0.011 (−0.093,0.071)	0.795	0.128 (0.049,0.207)	0.011	−0.021 (−0.127,0.085)	0.694	−0.031 (−0.164,0.102)	0.658
UPDRS3	−0.008 (−0.075,0.058)	0.806	0.181 (0.079,0.283)	0.005	−0.006 (−0.093,0.082)	0.901	0.054 (−0.072,0.181)	0.417
UPDRS4	0.012 (−0.067,0.091)	0.772	−0.045 (−0.085, −0.006)	0.058	0.029 (−0.073,0.130)	0.581	0.059 (−0.101,0.218)	0.492
MMSE	0.083 (−0.045,0.211)	0.203	0.136 (−0.130,0.402)	0.347	0.097 (−0.075,0.268)	0.269	0.099 (−0.105,0.304)	0.365
MoCA	0.086 (−0.366,0.538)	0.709	0.099 (−0.235,0.433)	0.586	−0.038 (−0.588,0.512)	0.892	−0.083 (−0.835,0.669)	0.835
SEADL	−0.176 (−0.824,0.472)	0.595	0.470 (−0.113,1.052)	0.148	0.115 (−0.701,0.931)	0.783	0.226 (−0.979,1.430)	0.721

Abbreviations: *HY3* Hoehn-Yahr stage of 3 or more, *RBD* Rapid eye movement sleep behavior disorder, *UPDRS* Unified Parkinson’s Disease Rating Scale, *MMSE* Mini–Mental State Examination, *MoCA* Montreal Cognitive Assessment, *SEADL* Schwab and England Activities of Daily Living Scale.

**Table 2:** Robust Mendelian randomization analysis of association between ganglion cell inner plexiform layer thickness and Parkinson’s disease.

	RAPS		MR-Egger		WME		Weighted MBE	
Outcome	Beta or OR (95%CI)	*P*	Beta or OR (95%CI)	*P*	Beta or OR (95%CI)	*P*	Beta or OR (95%CI)	*P*
Risk	1.006 (0.982,1.029)	0.641	1.029 (0.925,1.144)	0.606	1.014 (0.980,1.049)	0.436	1.016 (0.973,1.061)	0.482
Constipation	0.900 (0.771,1.051)	0.184	1.026 (0.727,1.446)	0.889	0.947 (0.773,1.159)	0.595	0.964 (0.742,1.253)	0.790
Daytime sleepiness	0.960 (0.823,1.120)	0.605	1.067 (0.754,1.510)	0.722	0.930 (0.751,1.150)	0.502	0.893 (0.653,1.220)	0.493
Dementia	1.076 (0.924,1.253)	0.348	0.991 (0.700,1.402)	0.960	1.061 (0.879,1.280)	0.537	1.039 (0.817,1.322)	0.759
Depression	1.104 (0.933,1.305)	0.249	1.185 (0.811,1.730)	0.403	1.059 (0.870,1.289)	0.569	1.056 (0.814,1.372)	0.689
Dyskinesias	1.089 (0.901,1.318)	0.377	1.059 (0.691,1.623)	0.800	0.988 (0.767,1.273)	0.929	0.946 (0.635,1.410)	0.794
HY3	1.059 (0.848,1.322)	0.613	1.056 (0.635,1.757)	0.838	1.120 (0.838,1.497)	0.445	1.138 (0.703,1.843)	0.611
Hyposmia	1.017 (0.881,1.174)	0.816	1.075 (0.774,1.493)	0.676	0.997 (0.825,1.204)	0.971	0.981 (0.745,1.292)	0.894
Insomnia	1.090 (0.963,1.234)	0.172	1.218 (1.027,1.445)	0.047	1.123 (0.959,1.315)	0.148	1.126 (0.910,1.393)	0.299
Motor fluctuation	0.982 (0.834,1.158)	0.832	1.244 (0.878,1.763)	0.266	0.962 (0.782,1.183)	0.713	0.936 (0.712,1.229)	0.648
RBD	1.199 (0.972,1.479)	0.091	1.461 (0.893,2.391)	0.166	1.328 (1.019,1.730)	0.036	1.354 (0.935,1.960)	0.140
AAO	−0.054 (−0.210,0.103)	0.503	−0.144 (−0.514,0.226)	0.455	−0.037 (−0.241,0.167)	0.722	−0.006 (−0.276,0.264)	0.968
UPDRS total	0.012 (−0.032,0.057)	0.592	−0.003 (−0.091,0.085)	0.951	0.038 (−0.021,0.097)	0.203	0.038 (−0.047,0.123)	0.397
UPDRS1	−0.011 (−0.064,0.041)	0.676	0.000 (−0.117,0.117)	0.999	0.001 (−0.065,0.067)	0.967	0.002 (−0.075,0.079)	0.963
UPDRS2	−0.023 (−0.076,0.031)	0.409	0.052 (−0.067,0.171)	0.415	−0.031 (−0.100,0.039)	0.391	−0.040 (−0.135,0.054)	0.427
UPDRS3	−0.022 (−0.068,0.024)	0.347	−0.068 (−0.170,0.035)	0.230	−0.015 (−0.076,0.045)	0.616	−0.011 (−0.096,0.073)	0.800
UPDRS4	−0.004 (−0.053,0.045)	0.865	0.025 (−0.037,0.087)	0.454	0.014 (−0.047,0.075)	0.654	0.019 (−0.047,0.084)	0.587
MMSE	0.009 (−0.082,0.101)	0.839	−0.019 (−0.185,0.147)	0.829	0.029 (−0.092,0.150)	0.641	0.042 (−0.097,0.182)	0.567
MoCA	−0.067 (−0.306,0.172)	0.581	0.060 (−0.452,0.572)	0.827	−0.209 (−0.509,0.092)	0.173	−0.242 (−0.689,0.205)	0.325
SEADL	−0.160 (−0.578,0.258)	0.453	0.028 (−0.728,0.783)	0.944	0.133 (−0.394,0.660)	0.622	0.151 (−0.551,0.854)	0.680

Abbreviations: *HY3* Hoehn-Yahr stage of 3 or more, *RBD* Rapid eye movement sleep behavior disorder, *UPDRS* Unified Parkinson’s Disease Rating Scale, *MMSE* Mini–Mental State Examination, *MoCA* Montreal Cognitive Assessment, *SEADL* Schwab and England Activities of Daily Living Scale.

### Association between genetically predicted RNFL and GCIPL thickness and PD AAO

There was no association between genetically predicted RNFL thickness (IVW *β* = −0.072, 95% CI (−0.321, 0.177), *P* = 0.572), GCIPL thickness (IVW *β* = −0.053, 95% CI (−0.174, 0.068), *P* = 0.389) and PD AAO (Fig. 2b). In the sensitivity analysis, the robust MR model found no correlation between RNFL or GCIPL thickness and PD AAO (Tables 1, 2). Regarding the quality control of IVs, all associations successfully passed the heterogeneity test (Supplementary Table 3) and the pleiotropic test (Supplementary Table 4). With regard to statistical power, our analysis is sufficiently equipped to detect a 20% alteration in PD AAO (Supplementary Table 5).

### Association between genetically predicted RNFL and GCIPL thickness and PD progression

Our MR study observed a statistically significant association between reduced RNFL thickness and reduced risk of constipation in PD (IVW OR = 0.854, 95% CI (0.782, 0.933) per SD, *P* < 0.001, FDR-corrected *P* = 0.018) (Fig. 2a). In addition, we also observed suggestive evidence for genetically predicted association between reduced RNFL thickness and decreased UPDRS total score (IVW β = −0.042, 95% CI (−0.079, −0.005) per SD, *P* = 0.025, FDR-corrected *P* = 0.180) (Fig. 2b), and suggestive evidence for genetically predicted association between reduced GCIPL thickness and reduced risk of constipation (IVW OR = 0.901, 95% CI (0.821, 0.988) per SD, *P* = 0.027, FDR-corrected *P* = 0.180), higher risk of depression (IVW OR = 1.103, 95% CI (1.016, 1.198) per SD, *P* = 0.020, FDR-corrected *P* = 0.180), higher risk of insomnia (IVW OR = 1.090, 95% CI (1.013, 1.172) per SD, *P* = 0.021, FDR-corrected *P* = 0.180), and higher risk of RBD (IVW OR = 1.198, 95% CI (1.061, 1.352) per SD, *P* = 0.003, FDR-corrected *P* = 0.069) (Fig. 2b). The estimated effect sizes of the SNPs on those exposure and PD outcomes were displayed in a scatter plot (Supplementary Figure 1). The LOO analysis did not identify any SNPs that had a significant impact on the estimated overall effect (Supplementary Figure 2).

**Fig. 3:**
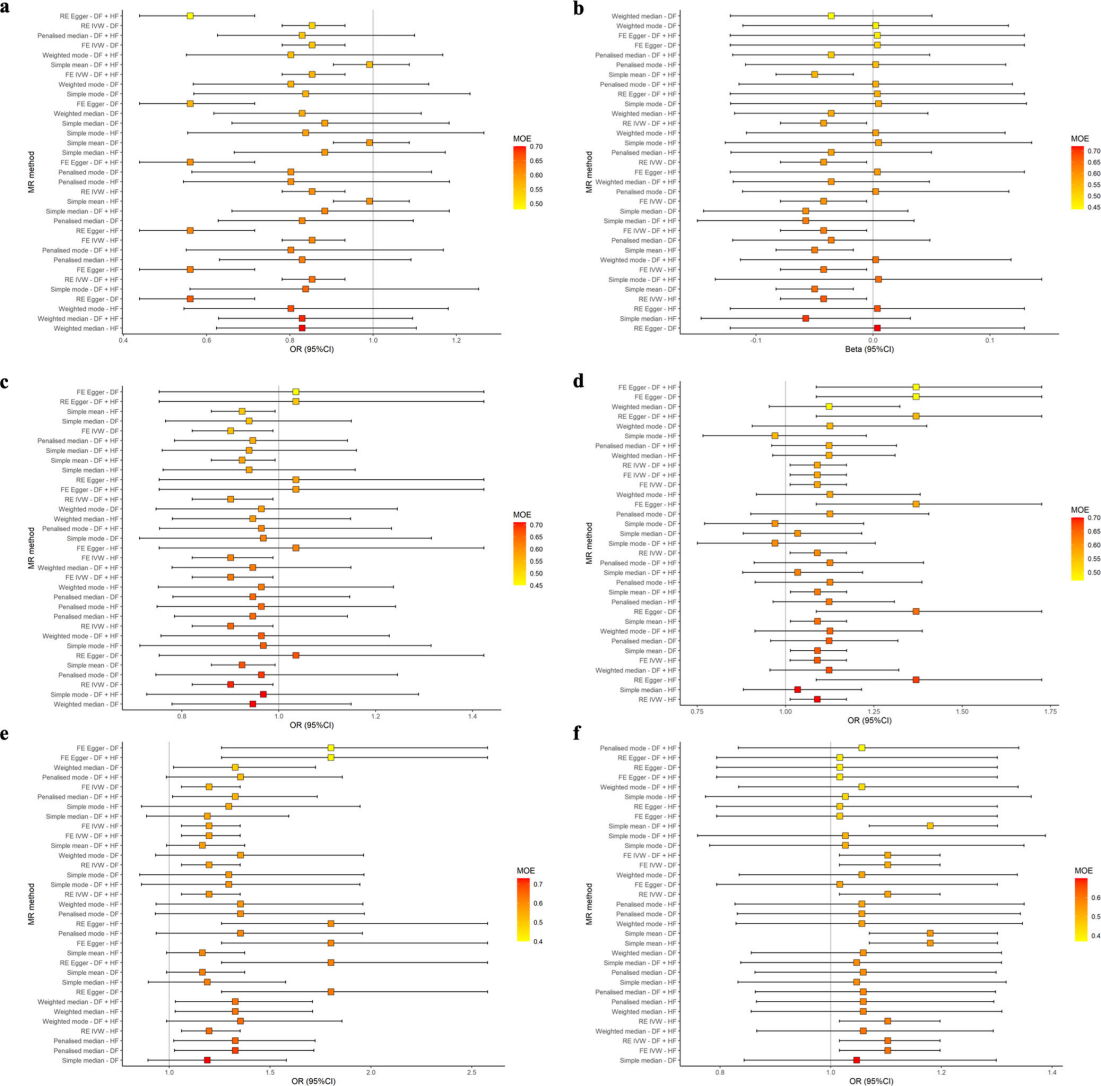
Mendelian randomization estimates from various methods using the Mixture of Experts approach. The Mendelian randomization (MR) estimates of (**a**) retinal nerve fiber layer (RNFL) thickness on constipation, (**b**) RNFL thickness on Unified Parkinson’s Disease Rating Scale (UPDRS) total scale, (**c**) ganglion cell inner plexiform layer (GCIPL) thickness on constipation, (**d**) GCIPL thickness on insomnia, (**e**) GCIPL thickness on rapid eye movement sleep behavior disorder (RBD), (**f**) GCIPL thickness on depression. The MR-MoE analysis indicates that apart from some inconsistencies in the model assessing the association between RNFL thickness and the UPRDS total score, and between GCIPL thickness and the risk of PD constipation and depression, the correlation between RNFL and GCIPL thickness and other PD phenotypes remains consistent. The vertical axis represents various combinations of the MR method and single nucleotide polymorphisms selection approaches. The estimates are colored and arranged according to the “Mixture of Experts (MOE)” statistics. MOE statistics nearing 1 suggest a greater probability that the specified MR method will yield a precise estimate for the specified dataset.

The sensitivity analysis revealed that the MR‒Egger result for the effect estimates of RNFL thickness with respect to the risk of constipation in PD was not in agreement with the IVW model (Table 1). However, the SNPs selected for the MR analysis of RNFL thickness with respect to the risk of constipation in PD showed a lower *I*^2^_(GX)_ statistic (0.76) (Supplementary Table 2), indicating that MR‒Egger might produce substantial bias. Notably, within the MR-MoE framework, all models, including MR‒Egger, generated effect estimates that were consistent with the IVW model (Fig. 3), implying that the correlation between decreased RNFL thickness and reduced risk of constipation in PD is robust. In the MR-MoE analysis of GCIPL thickness and PD depression risk, certain models deviate from the IVW model (Fig. 3). Notably, these models present smaller MoE values, indicative of their less optimal fit. The model with the highest MoE value yields an effect estimate aligned with the IVW model, strengthening our confidence in the robust association between GCIPL thickness and PD depression risk. Moreover, in the sensitivity analysis, it is observed that the robust MR models also align with the IVW model (Table 2), further reinforcing the robustness of this association. Additionally, both the robust MR model and the MR-MoE analysis suggested that the effect estimates for RNFL thickness on UPDRS total score **(**Table 1, Fig. 3) and for GCIPL thickness on PD-associated constipation risk (Table 2, Fig. 3) lacked robustness. This implies potential violations of core assumptions by some IVs. Nevertheless, the quality control assessment of IVs revealed no evidence of heterogeneity (Supplementary Table 3) or pleiotropy (Supplementary Table 4). Consequently, further evaluation of these associations is warranted. Post hoc statistical power calculations revealed a low level of statistical power, rendering it arduous to detect effect sizes below a 20% change in PD progression (Supplementary Table 5).

## Discussion

Utilizing the largest available GWAS datasets for PD, RNFL and GCIPL thickness, we performed a comprehensive two-sample MR analysis that provided evidence for an association between reduced RNFL and GCIPL thickness and nonmotor symptoms in PD. It is noteworthy that, similar to nonmotor symptoms, some evidence suggests that a decrease in retinal thickness may occur in the early stages of PD^41,42^. This provides chronological support for the association between retinal thinning and nonmotor symptoms of PD, as they also tend to occur early.

Constipation represents a significant challenge for patients with PD. Epidemiological data indicate that ~24.6–63% of individuals with PD experience constipation^43^. Furthermore, there exists a correlation between the severity of constipation and the severity of PD^44^. Crucially, constipation has the potential to impede the absorption of anti-PD drugs, resulting in fluctuations or diminished efficacy^45,46^. Concurrently, these medications might exacerbate constipation, thereby intensifying the issue^43^. Thus, proactively predicting and addressing the risk of constipation in patients with PD could potentially enhance the effectiveness of therapeutic interventions. Our study elucidates a substantial correlation between retinal thickness and the risk of constipation in patients with PD. To our knowledge, there have been no reported studies on the correlation between changes in retinal thickness and constipation symptoms in patients with PD. A cross-sectional study demonstrated a significant correlation between constipation and visual impairment in patients with PD; however, it did not investigate the potential relationship between retinal thickness and constipation symptoms^47^. We posit that the observed association between retinal thickness and constipation symptoms in individuals with PD may be attributed to circadian rhythms, melatonin, and gut microbiota. A reduction in retinal thickness is indicative of a diminution in retinal cells, potentially resulting in alterations to circadian rhythms and melatonin secretion. Studies have demonstrated that circadian rhythms play a crucial role in regulating defecation-related physiological processes, including gastrointestinal motility and intestinal permeability, with their disruption potentially resulting in gastrointestinal symptoms such as constipation and diarrhea^48,49^. Melatonin is a crucial hormone in modulating circadian rhythm^50^, and it has the potential to influence bowel habits via two distinct pathways. On one hand, melatonin can modulate gastrointestinal motility through regulating circadian rhythms; on the other hand, studies have found that there are high concentrations of melatonin in the gastrointestinal tract, which can directly regulate its motility^51^. The composition and function of the gut microbiota are intricately connected to bowel habits^52,53^. Significantly, studies have demonstrated that circadian rhythms and melatonin play a role in modulating the composition and function of the gut microbiota^54,55^. Furthermore, observational studies have provided evidence correlating retinal thickness with gut microbiota^56^, implying that gut microbiota may serve as a mediator between retinal degenerative changes and constipation. Nevertheless, the underlying mechanisms connecting retinal degeneration and symptoms of constipation in patients with PD necessitate additional investigation through animal and cellular models.

Sleep disorders are common nonmotor symptoms of PD, including insomnia and RBD. The present study provided suggestive statistical significance between the decrease in GCIPL thickness and higher risk of insomnia and RBD in patients with PD. Consistent with this study, previous observational studies have reported a correlation between the reduction of mRGCs and sleep disorders in PD patients^30^. mRGCs play a pivotal role in the regulation of circadian rhythms and melatonin secretion. A decrease in the number of mRGCs may precipitate disruptions in circadian rhythms and melatonin production, potentially culminating in sleep disorders. This provides a potential mechanism for the findings in this study. In addition, a decrease in retinal thickness among patients with RBD has also been observed in observational studies^42^. Yang et al. reported that PD patients with RBD had thinner RNFL thickness than PD patients without RBD. However, they did not evaluate the GCIPL thickness^57^. The present study provides further evidence of an association between reduced GCIPL thickness and sleep disorders in patients with PD, suggesting that GCIPL may be an effective target that can be used to improve sleep disorders in PD.

This study also observed a suggestive association between reduced GCIPL thickness and increased risk of depression in PD patients. An observational study reported an association between visual impairment and depressive symptoms in patients with PD^58^, but no study has yet reported a correlation between retinal thickness and depression symptoms in PD patients. Interestingly, observational studies have reported evidence of retinal thinning in patients with major depressive disorder, and reduced GCIPL thickness may be more closely associated with depression than RNFL thickness^59^. This is consistent with the evidence observed in this study that suggests an effect between GCIPL thickness, rather than RNFL thickness, and the risk of depression in PD.

We did not observe an association between the reduction in RNFL or GCIPL thickness and the progression of motor symptoms. Although our study results suggest that a reduction in RNFL thickness could potentially result in a reduction in the UPDRS total score, no significant association was observed between the reduction in RNFL thickness and the individual scores of UPDRS part I-IV. Consistent with the present study, a prospective cohort study carried out by Murueta-Goyena et al. demonstrated that reduced retinal thickness was linked to cognitive deterioration in patients with PD. However, the study did not reveal any correlation between retinal thickness and motor symptoms in patients with PD, indicating that the degeneration of retinal and motor-related brain regions is driven by distinct pathophysiological mechanisms^60^. However, further investigations are required to explore the association between alterations in retinal thickness and motor symptoms of PD.

There are some inherent limitations of our MR study. First, observational studies indicate that PD may only be associated with changes in RNFL and GCIPL thickness in specific quadrants rather than an overall change^25^. However, we were unable to obtain quadrant-specific summary data of RNFL and GCIPL thickness and therefore could not further perform subgroup analysis. Second, the statistical power of this study to detect changes in effect size associated with the progression of PD may be modest, and it is possible that smaller effect size changes may not have been discernible. Finally, there is a possibility of sample overlap between the GWAS summary data for RNFL and GCIPL thickness and PD risk. Nevertheless, the degree of bias associated with sample overlap is inversely proportional to the F-statistic^61^. In this study, the SNP set utilized had a larger mean F-statistic (42.78-61.48), thus suggesting that the sample overlap may not have influenced the results of this study^62^. Additionally, a recent study has indicated that main two-sample MR methods can be safely utilized for analyzing the UKB database without being affected by sample overlap^63^.

Using large GWAS datasets, our two sample MR study identify for the first time an association between the thinning of the RNFL and GCIPL and PD nonmotor symptoms. Changes in retinal thickness can potentially be explored as a reliable biomarker for monitoring the nonmotor symptom progression in PD. Further studies to examine the underlying pathophysiologic mechanisms of our observations may provide novel insights.

## Methods

### Acquisition of instrumental variables

The instrumental variables (IVs) utilized in this study were extracted from published GWASs. To avoid bias caused by population stratification, this study only utilized GWAS summary data from European populations.

### Acquisition of instrumental variables related to retinal thickness

The summary data on the SNPs associated with the thickness of RNFL or GCIPL were obtained derived from the GWAS meta-analysis by Currant et al.based on the United Kingdom Biobank (UK Biobank) database^39^. Specifically, the UK Biobank collected biological data from over 500,000 participants, aged 40 to 69, recruited across the UK between 2006 and 2010. Among these participants, 67,321 underwent an OCT examination. Currant et al. conducted genotyping and phenotype data quality control on these samples. The specific quality control process is detailed in the original publication. Ultimately, they selected a total of 31,434 datasets for the GWAS analysis of RNFL and GCIPL thickness. The associations between SNPs and retinal thickness were evaluated using an additive linear model while taking into account covariates such as eye-specific factors, technical factors, age, weight, height, and sex, among others.

A GWAS-significant threshold of *P* < 5.0 × 10^−8^ was used to initially identify SNPs associated with RNFL or GCIPL thickness. We computed the F-statistic for each SNP by utilizing the Eq. (1)^64^ and eliminated SNPs with an F-statistic below 10 to prevent the inclusion of weak SNPs^65^.

1 $$F=\frac{{R}^{2}\times (N-2)}{1-{R}^{2}}$$

Here, *R*^2^ represents the proportion of variance in RNFL or GCIPL thickness that can be attributed to the selected SNP, and N represents the sample size for GWAS on RNFL or GCIPL thickness.

Linkage disequilibrium (LD) refers to the tendency of adjacent genetic variations on a genome to be co-inherited^66^. Confounding factors might be introduced into the analysis by being in a state of high LD with selected genetic variations, potentially leading to bias. In this study, we employed stringent statistical parameters (*R*^*2*^ < 0.001, Window size > 10,000 kb)^67^ to conduct an LD analysis based on the 1000 Genomes Project’s European population reference panel^68^. We excluded variants in high LD to reduce potential bias.

Subsequently, we determined the effects of SNPs on the outcome phenotypes from the summary data of PD. To ensure alignment of effect alleles, the exposure and outcome datasets underwent harmonization, wherein all palindromic SNPs were excluded.

Steiger filtering is a statistical method designed to eliminate invalid IVs. The underlying principle of this method is that valid IVs should explain more variance in exposure than outcome traits. As a result, genetic variants that do not meet this criterion are discarded. By retaining genetic variation that explains a larger portion of the variance in exposure traits, Steiger filtering can effectively mitigates potential reverse causality effects^69^. To circumvent the potential reverse causality of PD resulting in retinal thinning in this study, we employed the Steiger filtering method to exclude SNPs that explain a greater variance in PD-related traits compared to retinal thickness.

### Acquisition of instrumental variables related to PD

This study primarily focuses on three aspects of PD assessment: risk of onset, age of onset, and disease progression. We used the GWAS summary data on PD onset risk from a 2019 meta-analysis conducted by Nalls et al.^36^ This comprehensive study incorporated 17 European PD cohorts, encompassing 37,688 PD patients (either self-reported or clinically diagnosed), 18,618 proxy cases from the UK Biobank (undetected but with a first-degree relative confirmed), and ~1.4 million control participants. The findings identified 90 genetic loci associated with PD onset risk. We extracted publicly available data for 33,647 patients with PD and 449,056 control participants from the Medical Research Council Integrative Epidemiology Unit (MRC IEU) OpenGWAS database^70^.

The PD AAO is pivotal for prognostic evaluations and forecasting disease trajectories. Blauwendraat et al.^38^ conducted a GWAS investigating the AAO for 28,568 PD patients from 17 cohorts from both International Parkinson Disease Genomics Consortium (IPDGC) and 23andMe (USA), identifying several genetic loci associated with PD AAO. PD AAO is defined by the age at which the initial symptoms emerge; if this is indeterminable, the age at PD diagnosis is used as a reference. Owing to the absence of publicly accessible data from 23andMe, we were able to procure AAO GWAS data for 17,996 PD patients publicly released by IPDGC.

The PD progression is defined by the severity of symptoms, using specific scales and the coexistence of other binary clinical indicators. Iwaki et al.^37^ conducted a GWAS analysis on 25 clinical phenotypes of 4,093 PD patients distributed across 12 cohorts. Acknowledging potential discrepancies in scales and binary outcome definitions among these cohorts, the researchers judiciously standardized these scores prior to analysis. For binary outcomes, a concerted effort was made to adopt a consistent definitional standard, with detailed criteria outlined in the supplemental material of the original publication. We collected the GWAS summary data of 18 clinical phenotypes among them, as shown in Supplementary Table 1. Specifically, these 18 clinical phenotypes include 10 binary outcomes: Hoehn-Yahr stage of 3 or more (H&Y3), motor fluctuations, dyskinesias, dementia, depression, rapid eye movement sleep behavior disorder (RBD), constipation, daytime sleepiness, insomnia, and hyposmia; and 8 continuous outcomes: Montreal Cognitive Assessment (MoCA), Mini-Mental State Examination (MMSE), Unified Parkinson’s Disease Rating Scale (UPDRS) I-IV & total and the modified Schwab and England Activities of Daily Living Scale (SEADL).

### Assessment of instrumental variables

Mendelian randomization requires the utilization of IVs that adhere to three essential assumptions to produce unbiased effect inference (1) The IVs must have a significant association with the exposure (relevance assumption). (2) There must be no association between IVs and any confounding factors (independence assumption). (3) The IVs should influence the outcome solely through their impact on the designated exposure (exclusion restriction assumption)^33^. In the present study, we employed subsequent procedures to scrutinize the essential assumptions.

Firstly, we conducted an evaluation of pleiotropy. Pleiotropy refers to the possibility that a SNP may be associated with multiple phenotypes. The presence of pleiotropic SNPs can violate the independence and exclusivity assumptions of instrumental variables, thereby introducing bias^32^. We used two conventional methods for assessing pleiotropy: MR-Egger intercept test^71^ and MR-PRESSO global test^72^. A *p*-value <0.05 in either the MR-Egger intercept test or the MR-PRESSO global test can be considered indicative of a pleiotropic effect.

Theoretically, effect estimates for outcomes should remain homogeneous among SNPs of the same exposure. If there is significant heterogeneity, it may indicate the presence of invalid SNPs^73^. To determine the extent of heterogeneity across effect estimates, we utilized both Cochran’s Q test and the *I*^2^ statistic. The indicator of heterogeneity was *p*-values of Cochran’s Q test below 0.05. The *I*^2^ statistic serves as a metric for quantifying the extent of heterogeneity. Generally, *I*^2^ values >25% indicate a substantial degree of heterogeneity^74^.

### Mendelian randomization analysis

We conducted the primary analysis using the Inverse Variance Weighted (IVW) method with multiplicative random effects. This method is optimal for two-sample MR, provided that SNPs strictly adhere to essential assumptions^75^. Our study involved forty independent statistical tests, with two exposures (RNFL and GCIPL thickness) tested across twenty distinct PD outcomes. To correct for multiple hypothesis testing, we utilized the false discovery rate (FDR) approach in this study^76^. Significance was determined as FDR-corrected *p*-values <0.05, whereas *p*-values <0.05 that did not meet the FDR-corrected threshold were regarded as suggestive evidence of an association. In addition, for the significant associations determined by the IVW model, we further conducted Mendelian randomization Mixture of Experts (MR-MoE) and leave-one-out (LOO) analyses to test the robustness of the results. MR-MoE harnesses machine learning algorithms to assess the optimal combination of various SNP selection strategies and MR models, thereby minimizing the influence of pleiotropic SNPs and maximizing the model’s statistical performance. MR-MoE calculates MoE values to quantify model performance, and MoE values range from 0 to 1, with 1 indicating that MR-MoE evaluates the best combination^77^. The LOO analysis iteratively removes a single SNP from the dataset and re-estimates its effect, allowing for the determination of any SNPs with significant impacts on the association.

### Power calculations

The post hoc power of our MR study was calculated by setting the expected odds ratio (OR) or beta coefficient. We calculated the power to detect an OR of 1.2 for each binomial outcome and a 0.2 standard deviation (SD) increase for each continuous outcome. The sample sizes for the progression of PD were determined by averaging the means of all SNPs in the GWAS summary statistics. The significance level for the two-tailed test was established as 0.05. An online calculator was utilized for the power calculation (https://sb452.shinyapps.io/power/).

### Sensitivity analysis

Given the constraints of MR due to IVs and model assumptions, we’ve employed various robust MR models for sensitivity analysis to bolster result reliability^78^. These models included MR‒Egger, Robust Adjusted Profile Score (RAPS), weighted median estimator (WME), and weighted mode-based estimate (MBE). These models are based on different IVs and model assumptions. If the effect estimates derived from different models are consistent, it indicates that the effect estimate is robust.

MR-Egger method—this method offers a relaxed requirement for exclusion restriction and accommodates pleiotropic effects in all SNPs under the assumption that Instrument Strength Independent of Direct Effect (INSIDE) holds^79^. Although the requirement of pleiotropic SNPs has been relaxed, the MR‒Egger model manifests reduced statistical power and heightened susceptibility to the bias of weak SNPs. The impact of weak SNPs on the effect estimates of MR‒Egger can be assessed by the regression dilution *I*^2^_(GX)_ statistic. If *I*^2^_(GX)_ falls below 0.9, it suggests significant bias from weak SNPs in the MR-Egger estimate, requiring SIMEX correction^80^. However, when *I*^2^_(GX)_ is below 0.6, even the SIMEX-corrected MR‒Egger estimator may introduce substantial bias, thus warranting cautious interpretation of the results^81^.

RAPS method—this method relaxes the requirements for the assumptions of relevance and exclusion restriction. When the model assumption of overall pleiotropy balance is satisfied, RAPS can generate unbiased estimations of effects even in the presence of weak or pleiotropic SNPs^82^.

Weighted median estimator method—this method assigns different weights to the estimates of the SNPs, assigning greater weight to more precise estimates. The method produces unbiased estimates if over 50% of the effect estimate weights originate from valid SNPs^83^.

Weighted mode-based estimate method—this method only requires the largest subset of SNPs with homogeneous effect estimates to be composed of valid SNPs to produce unbiased estimates^84^.

### Statistical software

The analyses were conducted with R version 4.2.0, utilizing several primary R packages. These packages included TwoSampleMR (version 0.5.6)^67^, MRPRESSO (version 1.0)^72^, mr.raps (version 0.2)^82^, and simex (version 1.8)^80^.

### Supplementary information

Supplemental material

Related Manuscript File

## Data Availability

The datasets supporting the conclusions of this article are available in the public repository. GWAS summary data on RNFL and GCIPL thickness can be obtained from the GWAS Catalog (https://www.ebi.ac.uk/gwas/). Summary data on PD risk can be obtained from the Medical Research Council Integrative Epidemiology Unit (MRC-IEU) OpenGWAS (https://gwas.mrcieu.ac.uk/). Summary data on PD progression can be obtained from the International Parkinson Disease Genomics Consortium (IPDGC) (https://pdgenetics.org/resources) database.

## Code availability

The code or algorithms employed for result generation in this study can be obtained from the corresponding authors upon a justified request.
