# Supplementary material for: Mendelian randomization reveals association between retinal thickness and non-motor symptoms of Parkinson’s disease
Source: NPJ Parkinsons Dis. 2023 Dec 13;9:163. doi: 10.1038/s41531-023-00611-z (PMC10719335; doi:10.1038/s41531-023-00611-z)
Supplement: Supplementary file 1 — Supplemental material [file 41531_2023_611_MOESM1_ESM.pdf]

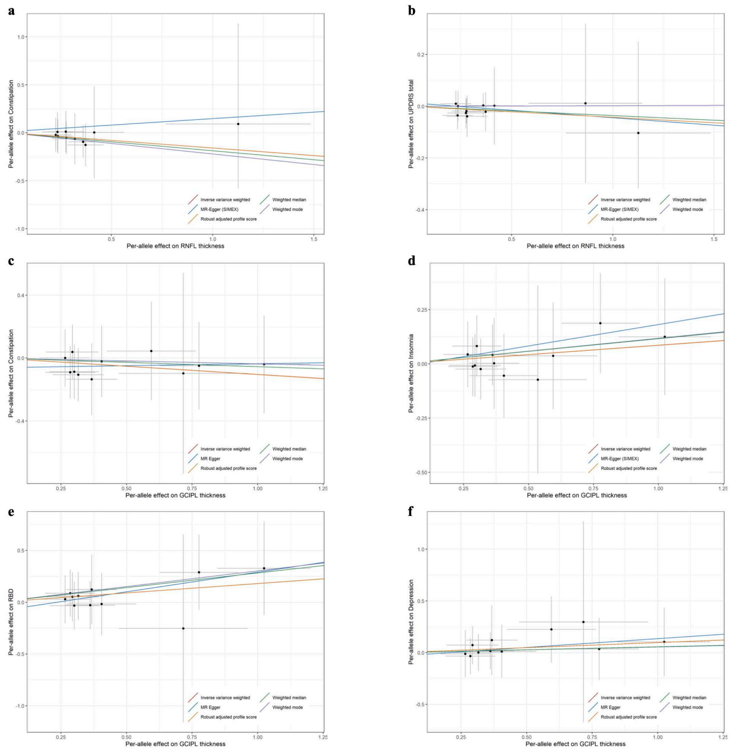

Supplementary Figure 1 Scatter plot of Mendelian randomization study on the association between retinal thickness and Parkinson's disease

Scatter plot showing the effects of single nucleotide polymorphisms on retinal nerve fiber layer (RNFL) thickness or ganglion cell inner plexiform layer (GCIPL) thickness against their effects on Parkinson’s disease (PD) outcomes. The model fit lines indicate Mendelian randomization (MR) estimates from different MR methods. (a) RNFL thickness against constipation, (b) RNFL thickness against Unified Parkinson’s Disease Rating Scale (UPDRS) total scale, (c) GCIPL thickness against constipation, (d) GCIPL thickness against insomnia, (e) GCIPL thickness against rapid eye movement sleep behavior disorder, (f) GCIPL thickness against depression

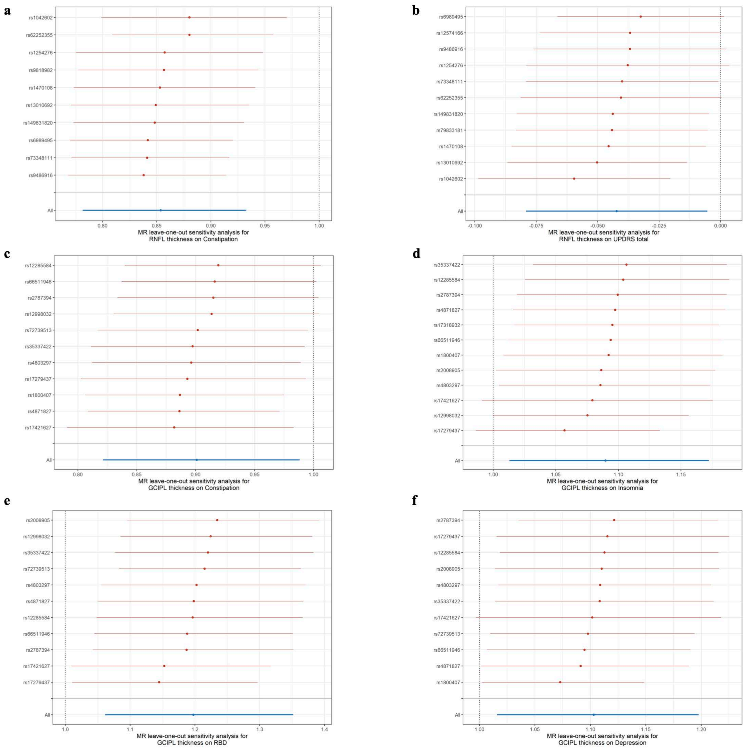

Supplementary Figure 2 Leave-one-out plot of the Mendelian randomization study on the association between retinal thickness and Parkinson's disease

Leave-one-out approach to identify potentially influential single nucleotide polymorphisms that exert a significant impact on association. Association between (a) retinal nerve fiber layer (RNFL) thickness and constipation, (b) RNFL thickness and Unified Parkinson’s Disease Rating Scale (UPDRS) total scale, (c) ganglion cell inner plexiform layer (GCIPL) thickness and constipation, (d) GCIPL thickness and insomnia, (e) GCIPL thickness and rapid eye movement sleep behavior disorder (RBD), and (f) GCIPL thickness and depression.

Supplementary Table 1 The information related to exposure and outcome phenotype used in this study

Trait	Variable type	Sample Size	Population	References
**Exposure**				
RNFL thickness	Continuous	31,434	Europeans	Currant et al[1]
GCIPL thickness	Continuous	31,434	Europeans	Currant et al[1]
**Outcome**				
PD Risk at onset	Binomial	482,730	Europeans	Nalls et al[2]
PD Age at onset	Continuous	17,996	Europeans	Blauwendraat et al[3]
PD Progression		4093	Europeans	Iwaki et al[4]
HY3	Binomial			
Motor fluctuations	Binomial			
Dyskinesias	Binomial			
Dementia	Binomial			
Depression	Binomial			
RBD	Binomial			
Constipation	Binomial			
Daytime sleepiness	Binomial			
Insomnia	Binomial			
Hyposmia	Binomial			
MoCA	Continuous			
MMSE	Continuous			
UPDRS I-IV & total	Continuous			
Modified SEADL	Continuous			

Abbreviations: RNFL, Retinal nerve fibre layer; GCIPL, Ganglion cell inner plexiform layer; HY3, Hoehn-Yahr stage of 3 or more; RBD, Rapid eye movement sleep behaviour disorder; UPDRS, Unified Parkinson's Disease Rating Scale; MMSE, Mini–Mental State Examination; MoCA, Montreal Cognitive Assessment; SEADL, Schwab and England Activities of Daily Living Scale.

Supplementary Table 2 Correlation assessment of instrumental variables with the thickness of the retinal nerve fiber layer and the ganglion cell inner plexiform layer

Exposure	Outcome	No. SNPs	PVE	mean F-Statistic (min, max)	$I^2_{(GX)}$
RNFL thickness	Risk	21	0.0359	53.87(29.90,115.48)	0.84
RNFL thickness	Constipation	10	0.0147	46.22(30.99, 92.98)	0.76
RNFL thickness	Daytime sleepiness	12	0.0184	48.18(29.90,109.13)	0.85
RNFL thickness	Dementia	13	0.0206	49.90(30.69,109.13)	0.83
RNFL thickness	Depression	11	0.0156	44.74(29.90, 92.98)	0.78
RNFL thickness	Dyskinesias	7	0.0113	50.67(30.99, 92.98)	0.59
RNFL thickness	HY3	9	0.0136	47.62(29.90, 92.98)	0.82
RNFL thickness	Hyposmia	11	0.0184	52.72(30.99,109.13)	0.84
RNFL thickness	Insomnia	14	0.0215	48.47(30.69,109.13)	0.83
RNFL thickness	Motor fluctuation	8	0.0125	49.14(32.52, 92.98)	0.50
RNFL thickness	RBD	8	0.0140	55.15(29.90,109.13)	0.86
RNFL thickness	AAO	16	0.0237	46.69(30.54,115.48)	0.77
RNFL thickness	UPDRS total	11	0.0158	45.17(30.69, 92.98)	0.81
RNFL thickness	UPDRS1	12	0.0168	44.12(30.69, 92.98)	0.80
RNFL thickness	UPDRS2	11	0.0157	44.81(30.69, 92.98)	0.74
RNFL thickness	UPDRS3	14	0.0214	48.08(29.90,109.13)	0.80
RNFL thickness	UPDRS4	9	0.0136	47.47(30.69, 92.98)	0.77
RNFL thickness	MMSE	10	0.0146	46.11(30.69, 92.98)	0.83
RNFL thickness	MoCA	7	0.0095	42.78(30.69, 67.96)	0.79
RNFL thickness	SEADL	11	0.0156	44.71(29.90, 92.98)	0.77
GCIPL thickness	Risk	21	0.0353	52.96(30.55,127.50)	0.86
GCIPL thickness	Constipation	11	0.0184	52.66(30.55,127.50)	0.91
GCIPL thickness	Daytime sleepiness	11	0.0189	54.17(30.55,127.50)	0.91
GCIPL thickness	Dementia	12	0.0194	50.92(30.55,127.50)	0.90
GCIPL thickness	Depression	11	0.0189	54.21(30.55,127.50)	0.90
GCIPL thickness	Dyskinesias	9	0.0163	57.00(30.55,127.50)	0.92
GCIPL thickness	HY3	10	0.0172	54.14(30.95,127.50)	0.91
GCIPL thickness	Hyposmia	11	0.0184	52.58(30.55,127.50)	0.90
GCIPL thickness	Insomnia	12	0.0201	52.82(30.55,127.50)	0.89
GCIPL thickness	Motor fluctuation	8	0.0156	61.48(30.55,127.50)	0.93
GCIPL thickness	RBD	11	0.0187	53.48(30.55,127.50)	0.90
GCIPL thickness	AAO	21	0.0353	52.96(30.55,127.50)	0.86
GCIPL thickness	UPDRS total	12	0.0197	51.60(30.55,127.50)	0.89
GCIPL thickness	UPDRS1	11	0.0184	52.58(30.55,127.50)	0.90
GCIPL thickness	UPDRS2	10	0.0174	54.75(30.55,127.50)	0.91
GCIPL thickness	UPDRS3	11	0.0190	54.49(30.55,127.50)	0.91
GCIPL thickness	UPDRS4	12	0.0201	52.66(30.55,127.50)	0.90
GCIPL thickness	MMSE	9	0.0141	49.18(30.55,127.50)	0.89
GCIPL thickness	MoCA	8	0.0152	59.71(30.55,127.50)	0.93
GCIPL thickness	SEADL	13	0.0212	51.27(30.55,127.50)	0.89

Abbreviations: SNP, Single nucleotide polymorphism; PVE, Proportion of phenotypic Variance Explained; RNFL, Retinal nerve fibre layer; GCIPL, Ganglion cell inner plexiform layer; HY3, Hoehn-Yahr stage of 3 or more; RBD, Rapid eye movement sleep behaviour disorder; AAO, Age at Onset; UPDRS, Unified Parkinson’s Disease Rating Scale; MMSE, Mini–Mental State Examination; MoCA, Montreal Cognitive Assessment; SEADL, Schwab and England Activities of Daily Living Scale

Supplementary Table 3 Heterogeneity test for association estimates of retinal nerve fiber layer and ganglion cell inner plexiform layer thickness and Parkinson's disease

Exposure	Outcome	No. SNPs	Q	DF	P	I^2
RNFL thickness	Risk	21	26.30	20	0.16	0.24
RNFL thickness	Constipation	10	1.39	9	1.00	0.00
RNFL thickness	Daytime sleepiness	12	3.83	11	0.97	0.00
RNFL thickness	Dementia	13	9.36	12	0.67	0.00
RNFL thickness	Depression	11	4.57	10	0.92	0.00
RNFL thickness	Dyskinesias	7	2.49	6	0.87	0.00
RNFL thickness	HY3	9	4.87	8	0.77	0.00
RNFL thickness	Hyposmia	11	3.11	10	0.98	0.00
RNFL thickness	Insomnia	14	6.30	13	0.93	0.00
RNFL thickness	Motor fluctuation	8	4.10	7	0.77	0.00
RNFL thickness	RBD	8	1.96	7	0.96	0.00
RNFL thickness	AAO	16	15.63	15	0.41	0.04
RNFL thickness	UPDRS total	11	3.20	10	0.98	0.00
RNFL thickness	UPDRS1	12	3.30	11	0.99	0.00
RNFL thickness	UPDRS2	11	2.89	10	0.98	0.00
RNFL thickness	UPDRS3	14	8.60	13	0.80	0.00
RNFL thickness	UPDRS4	9	2.85	8	0.94	0.00
RNFL thickness	MMSE	10	7.04	9	0.63	0.00
RNFL thickness	MoCA	7	3.14	6	0.79	0.00
RNFL thickness	SEADL	11	6.10	10	0.81	0.00
GCIPL thickness	Risk	21	32.16	20	0.04	0.38
GCIPL thickness	Constipation	11	3.76	10	0.96	0.00
GCIPL thickness	Daytime sleepiness	11	6.95	10	0.73	0.00
GCIPL thickness	Dementia	12	5.01	11	0.93	0.00
GCIPL thickness	Depression	11	2.55	10	0.99	0.00
GCIPL thickness	Dyskinesias	9	4.12	8	0.85	0.00
GCIPL thickness	HY3	10	4.06	9	0.91	0.00
GCIPL thickness	Hyposmia	11	7.35	10	0.69	0.00
GCIPL thickness	Insomnia	12	4.03	11	0.97	0.00
GCIPL thickness	Motor fluctuation	8	3.78	7	0.80	0.00
GCIPL thickness	RBD	11	3.49	10	0.97	0.00
GCIPL thickness	AAO	21	12.36	20	0.90	0.00
GCIPL thickness	UPDRS total	12	7.29	11	0.77	0.00
GCIPL thickness	UPDRS1	11	6.62	10	0.76	0.00
GCIPL thickness	UPDRS2	10	6.21	9	0.72	0.00
GCIPL thickness	UPDRS3	11	7.52	10	0.68	0.00
GCIPL thickness	UPDRS4	12	3.20	11	0.99	0.00
GCIPL thickness	MMSE	9	6.37	8	0.61	0.00
GCIPL thickness	MoCA	8	3.89	7	0.79	0.00
GCIPL thickness	SEADL	13	5.41	12	0.94	0.00

Abbreviations: SNP, Single nucleotide polymorphism; RNFL, Retinal nerve fibre layer; GCIPL, Ganglion cell inner plexiform layer; HY3, Hoehn-Yahr stage of 3 or more; RBD, Rapid eye movement sleep behaviour disorder; AAO, Age at Onset; UPDRS, Unified Parkinson’s Disease Rating Scale; MMSE, Mini–Mental State Examination; MoCA, Montreal Cognitive Assessment; SEADL, Schwab and England Activities of Daily Living Scale

Supplementary Table 4 Pleiotropy tests for association estimates of retinal nerve fiber layer and ganglion cell inner plexiform layer thickness and Parkinson's disease

		MR-Egger		MR-PRESSO global test	
Exposure	Outcome	Intercept (95%CI)	P	RSSobs	P
RNFL thickness	Risk	-0.033(-0.067,0.000)	0.068	28.901	0.158
RNFL thickness	Constipation	0.018(-0.241,0.277)	0.896	1.771	0.998
RNFL thickness	Daytime sleepiness	-0.006(-0.241,0.229)	0.962	5.076	0.961
RNFL thickness	Dementia	0.078(-0.164,0.320)	0.541	11.358	0.657
RNFL thickness	Depression	0.101(-0.180,0.382)	0.499	5.440	0.921
RNFL thickness	Dyskinesias	-0.156(-0.633,0.320)	0.549	3.432	0.870
RNFL thickness	HY3	-0.202(-0.540,0.137)	0.281	6.028	0.781
RNFL thickness	Hyposmia	-0.020(-0.287,0.247)	0.885	4.627	0.961
RNFL thickness	Insomnia	0.081(-0.103,0.266)	0.404	7.424	0.933
RNFL thickness	Motor fluctuation	0.071(-0.276,0.418)	0.702	5.703	0.754
RNFL thickness	RBD	0.040(-0.352,0.431)	0.849	2.562	0.962
RNFL thickness	AAO	0.034(-0.237,0.304)	0.811	18.043	0.404
RNFL thickness	UPDRS total	-0.010(-0.082,0.062)	0.800	4.131	0.973
RNFL thickness	UPDRS1	-0.021(-0.121,0.080)	0.696	3.984	0.985
RNFL thickness	UPDRS2	-0.010(-0.116,0.096)	0.861	3.459	0.987
RNFL thickness	UPDRS3	-0.040(-0.115,0.034)	0.308	10.299	0.799
RNFL thickness	UPDRS4	0.047(-0.055,0.150)	0.395	5.108	0.872
RNFL thickness	MMSE	0.000(-0.136,0.135)	0.997	8.669	0.656
RNFL thickness	MoCA	-0.102(-0.582,0.378)	0.694	4.309	0.788
RNFL thickness	SEADL	0.014(-0.698,0.727)	0.970	7.705	0.781
GCIPL thickness	Risk	-0.031(-0.063,0.002)	0.083	34.748	0.055
GCIPL thickness	Constipation	-0.061(-0.207,0.085)	0.432	4.469	0.960
GCIPL thickness	Daytime sleepiness	-0.051(-0.203,0.101)	0.527	9.157	0.687
GCIPL thickness	Dementia	0.038(-0.108,0.183)	0.621	5.672	0.947
GCIPL thickness	Depression	-0.034(-0.199,0.130)	0.691	2.978	0.994
GCIPL thickness	Dyskinesias	0.013(-0.172,0.199)	0.891	6.370	0.774
GCIPL thickness	HY3	0.001(-0.229,0.231)	0.993	6.273	0.837
GCIPL thickness	Hyposmia	-0.026(-0.164,0.112)	0.722	9.232	0.676
GCIPL thickness	Insomnia	-0.046(-0.167,0.076)	0.479	4.915	0.965
GCIPL thickness	Motor fluctuation	-0.121(-0.279,0.038)	0.186	5.938	0.735
GCIPL thickness	RBD	-0.088(-0.287,0.111)	0.408	4.447	0.963
GCIPL thickness	AAO	0.004(-0.177,0.185)	0.967	13.589	0.902
GCIPL thickness	UPDRS total	0.017(-0.025,0.060)	0.444	10.385	0.679
GCIPL thickness	UPDRS1	-0.005(-0.057,0.046)	0.840	7.704	0.785
GCIPL thickness	UPDRS2	-0.036(-0.088,0.016)	0.208	8.193	0.685
GCIPL thickness	UPDRS3	0.022(-0.022,0.066)	0.358	9.545	0.662
GCIPL thickness	UPDRS4	-0.010(-0.060,0.039)	0.690	4.105	0.987
GCIPL thickness	MMSE	0.018(-0.070,0.106)	0.700	7.824	0.631
GCIPL thickness	MoCA	-0.066(-0.305,0.173)	0.606	6.377	0.705
GCIPL thickness	SEADL	-0.129(-0.543,0.286)	0.555	6.653	0.935

Abbreviations: MR-PRESSO, Mendelian Randomisation Pleiotropy RESidual Sum and Outlier; RNFL, Retinal nerve fibre layer; GCIPL, Ganglion cell inner plexiform layer; HY3, Hoehn-Yahr stage of 3 or more; RBD, Rapid eye movement sleep behaviour disorder; AAO, Age at Onset; UPDRS, Unified Parkinson's Disease Rating Scale; MMSE, Mini–Mental State Examination; MoCA, Montreal Cognitive Assessment; SEADL, Schwab and England Activities of Daily Living Scale

Supplementary Table 5 Power calculations for Mendelian randomization study of association between retinal thickness and Parkinson's disease

Exposure	Outcome	No. SNPs	Power#
RNFL thickness	Risk	21	100.0%
RNFL thickness	Constipation	10	9.7%
RNFL thickness	Daytime sleepiness	12	11.1%
RNFL thickness	Dementia	13	13.0%
RNFL thickness	Depression	11	10.7%
RNFL thickness	Dyskinesias	7	7.9%
RNFL thickness	HY3	9	8.8%
RNFL thickness	Hyposmia	11	10.2%
RNFL thickness	Insomnia	14	13.2%
RNFL thickness	Motor fluctuation	8	9.5%
RNFL thickness	RBD	8	7.5%
RNFL thickness	AAO	16	98.5%
RNFL thickness	UPDRS total	11	24.1%
RNFL thickness	UPDRS1	12	20.1%
RNFL thickness	UPDRS2	11	19.9%
RNFL thickness	UPDRS3	14	26.5%
RNFL thickness	UPDRS4	9	15.7%
RNFL thickness	MMSE	10	16.8%
RNFL thickness	MoCA	7	9.1%
RNFL thickness	SEADL	11	19.6%
GCIPL thickness	Risk	21	100.0%
GCIPL thickness	Constipation	11	11.1%
GCIPL thickness	Daytime sleepiness	11	11.8%
GCIPL thickness	Dementia	12	12.0%
GCIPL thickness	Depression	11	12.2%
GCIPL thickness	Dyskinesias	9	10.1%
GCIPL thickness	HY3	10	10.0%
GCIPL thickness	Hyposmia	11	11.6%
GCIPL thickness	Insomnia	12	12.9%
GCIPL thickness	Motor fluctuation	8	10.6%
GCIPL thickness	RBD	11	8.9%
GCIPL thickness	AAO	21	99.9%
GCIPL thickness	UPDRS total	12	28.7%
GCIPL thickness	UPDRS1	11	23.9%
GCIPL thickness	UPDRS2	10	23.9%
GCIPL thickness	UPDRS3	11	27.4%
GCIPL thickness	UPDRS4	12	18.4%
GCIPL thickness	MMSE	9	18.0%
GCIPL thickness	MoCA	8	12.2%
GCIPL thickness	SEADL	13	24.0%

Power to detect an OR of 1.2 for each binomial outcome or a 0.2 SD increase for each continuous outcome.

Abbreviations: SNP, Single nucleotide polymorphism; PVE, Proportion of phenotypic Variance Explained; RNFL, Retinal nerve fibre layer; GCIPL, Ganglion cell inner plexiform layer; HY3, Hoehn-Yahr stage of 3 or more; RBD, Rapid eye movement sleep behaviour disorder; AAO, Age at Onset; UPDRS, Unified Parkinson’s Disease Rating Scale; MMSE, Mini–Mental State Examination; MoCA, Montreal Cognitive Assessment; SEADL, Schwab and England Activities of Daily Living Scale

Supplementary Table 6 STROBE-MR checklist of recommended items to address in reports of Mendelian randomization studies [5,6]

Item No.	Section	Checklist item	Page No.	Relevant text from manuscript
1	TITLE and ABSTRACT	Indicate Mendelian randomization (MR) as the study's design in the title and/or the abstract if that is a main purpose of the study	1,2	The term "Mendelian randomization" was incorporated into both the title and abstract.
	INTRODUCTION			
2	Background	Explain the scientific background and rationale for the reported study. What is the exposure? Is a potential causal relationship between exposure and outcome plausible? Justify why MR is a helpful method to address the study question	3,4	Introduction, Paragraph 1-4
3	Objectives	State specific objectives clearly, including pre-specified causal hypotheses (if any). State that MR is a method that, under specific assumptions, intends to estimate causal effects	4,5	Introduction, Paragraph 5
	METHODS			
4	Study design and data sources	Present key elements of the study design early in the article. Consider including a table listing sources of data for all phases of the study. For each data source contributing to the analysis, describe the following:		
	a)	Setting: Describe the study design and the underlying population, if possible. Describe the setting, locations, and relevant dates, including periods of recruitment, exposure, follow-up, and data collection, when available.	12	Method, Section "Acquisition of instrumental variables", Paragraph 1. Figure 1 depicts the flowchart of this study.
	b)	Participants: Give the eligibility criteria, and the sources and methods of selection of participants. Report the sample size, and whether any power or sample size calculations were carried out prior to the main analysis	12-15,17	Method, Information regarding the GWAS has been presented in the sections "Acquisition of instrumental variables related to retinal thickness" and "Acquisition of instrumental variables related to PD".<br>Furthermore, Table S1 also provides the Information about the GWAS on exposure and outcome in this study.<br>The section "Power calculations" provides the method for calculating statistical power in this study.
	c)	Describe measurement, quality control and selection of genetic variants	12-15	Method, Sections "Acquisition of instrumental

				variables related to retinal thickness" and "Acquisition of instrumental variables related to PD"
	d)	For each exposure, outcome, and other relevant variables, describe methods of assessment and diagnostic criteria for diseases	12-15	Method, the association analysis model and adjusted covariates for the GWAS study on retinal thickness are described in the section "Acquisition of instrumental variables related to retinal thickness'. The measurement method of Parkinson's disease-related outcome phenotypes is briefly described in the section "Acquisition of instrumental variables related to PD", and more specific explanations can be found in the original GWAS study.
	e)	Provide details of ethics committee approval and participant informed consent, if relevant	N/A	The summary data utilized in this study were extracted from published GWAS that had already passed ethical review. Hence, this study does not require additional ethical approval.
5	Assumptions	Explicitly state the three core IV assumptions for the main analysis (relevance, independence and exclusion restriction) as well assumptions for any additional or sensitivity analysis	15.18	Method, Section "Assessment of instrumental variables" and "Sensitivity Analysis"
6	Statistical methods: main analysis	Describe statistical methods and statistics used		
	a)	Describe how quantitative variables were handled in the analyses (i.e., scale, units, model)	N/A	
	b)	Describe how genetic variants were handled in the analyses and, if applicable, how their weights were selected	12-16	Method, In the sections " Acquisition of instrumental variables" and "Assessment of instrumental variables", we described the process of manipulating genetic variations.
	c)	Describe the MR estimator (e.g. two-stage least squares, Wald ratio) and related statistics. Detail the included covariates and, in case of two-sample MR, whether the same covariate set was used for adjustment in the two samples	16-19	Method, We described the details of the MR estimator and related statistics used in this study in the sections on " Mendelian randomization analysis "and "Sensitivity Analysis"
	d)	Explain how missing data were addressed	N/A	
	e)	If applicable, indicate how multiple testing was addressed	17	"To correct for multiple hypothesis testing, we utilized the false discovery

				rate (FDR) approach in this study"
7	Assessment of assumptions	Describe any methods or prior knowledge used to assess the assumptions or justify their validity	15-16	This study used Cochran's Q test and I^2 statistic for heterogeneity assessment, and MR-PRESSO for pleiotropy evaluation. We provided a detailed description in the section "Assessment of instrumental variables".
8	Sensitivity analyses and additional analyses	Describe any sensitivity analyses or additional analyses performed (e.g. comparison of effect estimates from different approaches, independent replication, bias analytic techniques, validation of instruments, simulations)	18-19	Method, Section "Sensitivity Analysis"
9	Software and pre-registration			
	a)	Name statistical software and package(s), including version and settings used	20	Method, The analyses were conducted with R version 4.2.0, utilizing several primary R packages. These packages included TwoSampleMR (version 0.5.6), MRPRESSO (version 1.0), mr.raps (version 0.2), and simex (version 1.8)
	b)	State whether the study protocol and details were pre-registered (as well as when and where)	N/A	
	RESULTS			
10	Descriptive data			
	a)	Report the numbers of individuals at each stage of included studies and reasons for exclusion. Consider use of a flow diagram	N/A	
	b)	Report summary statistics for phenotypic exposure(s), outcome(s), and other relevant variables (e.g. means, SDs, proportions)	N/A	
	c)	If the data sources include meta-analyses of previous studies, provide the assessments of heterogeneity across these studies	N/A	
	d)	For two-sample MR:<br>i. Provide justification of the similarity of the genetic variant-exposure associations between the exposure and outcome samples<br>ii. Provide information on the number of individuals who overlap between the exposure and outcome studies	12	Discussion, in this study, there may be some overlap between the GWAS samples of retinal thickness and PD risk, but we cannot assess it specifically. However, the F-statistic in our study is large, so it is unlikely that this sample overlap will affect the results of our study.

**11**	**Main results**			
	**a)**	**Report the associations between genetic variant and exposure, and between genetic variant and outcome, preferably on an interpretable scale**	**Table S2**	
	**b)**	**Report MR estimates of the relationship between exposure and outcome, and the measures of uncertainty from the MR analysis, on an interpretable scale, such as odds ratio or relative risk per SD difference**	**Figure 2**	
	**c)**	**If relevant, consider translating estimates of relative risk into absolute risk for a meaningful time period**	**N/A**	
	**d)**	**Consider plots to visualize results (e.g. forest plot, scatterplot of associations between genetic variants and outcome versus between genetic variants and exposure)**	**Figure 2 and Figure S1**	
**12**	**Assessment of assumptions**			
	**a)**	**Report the assessment of the validity of the assumptions**	**Table S3 and S4**	
	**b)**	**Report any additional statistics (e.g., assessments of heterogeneity across genetic variants, such as I2, Q statistic or E-value)**	**Table S3 and S4**	
**13**	**Sensitivity analyses and additional analyses**			
	**a)**	**Report any sensitivity analyses to assess the robustness of the main results to violations of the assumptions**	**Table 1 and 2; Figure 3 and S2**	
	**b)**	**Report results from other sensitivity analyses or additional analyses**	**Table 1 and 2; Figure 3 and S2**	
	**c)**	**Report any assessment of direction of causal relationship (e.g., bidirectional MR)**	**14**	**Methods, "To circumvent the potential reverse causality of PD resulting in retinal thinning in this study, we employed the Steiger filtering method to exclude SNPs that explain a greater variance in PD-related traits compared to retinal thickness."**
	**d)**	**When relevant, report and compare with estimates from non-MR analyses**	**N/A**	
	**e)**	**Consider additional plots to visualize results (e.g., leave-one-out analyses)**	**Figure S2**	
	**DISCUSSION**			

**14**	**Key results**	**Summarize key results with reference to study objectives**	**8**	**Discussion, "Utilizing the largest available GWAS datasets for PD, RNFL and GCIPL thickness, we performed a comprehensive two-sample MR analysis that provided evidence for an association between reduced RNFL and GCIPL thickness and nonmotor symptoms in PD."**
**15**	**Limitations**	**Discuss limitations of the study, taking into account the validity of the IV assumptions, other sources of potential bias, and imprecision. Discuss both direction and magnitude of any potential bias and any efforts to address them**	**11,12**	**Discussion, Paragraph 6**
**16**	**Interpretation**			
	**a)**	**Meaning: Give a cautious overall interpretation of results in the context of their limitations and in comparison with other studies**	**8-11**	**Discussion, Paragraph 2-5**
	**b)**	**Mechanism: Discuss underlying biological mechanisms that could drive a potential causal relationship between the investigated exposure and the outcome, and whether the gene-environment equivalence assumption is reasonable. Use causal language carefully, clarifying that IV estimates may provide causal effects only under certain assumptions**	**8-11**	**Discussion, Paragraph 2-5, E.g. "implying that gut microbiota may serve as a mediator between retinal degenerative changes and constipation"**
	**c)**	**Clinical relevance: Discuss whether the results have clinical or public policy relevance, and to what extent they inform effect sizes of possible interventions**	**8-11**	**Discussion, Paragraph 2-5, E.g. "The present study provides further evidence of a causal association between reduced GCIPL thickness and sleep disorders in patients with PD, suggesting that GCIPL may be an effective target that can be used to improve sleep disorders in PD"；"mRGCs play a pivotal role in the regulation of circadian rhythms and melatonin secretion. A decrease in the number of mRGCs may precipitate disruptions in circadian rhythms and melatonin production, potentially culminating in sleep disorders."**
**17**	**Generalizability**	**Discuss the generalizability of the study results (a) to other populations, (b) across other exposure periods/timings, and (c) across other levels of exposure**	**8-11**	**Discussion, Paragraph 2-5**
	**OTHER INFORMATION**			

**18**	**Funding**	**Describe sources of funding and the role of funders in the present study and, if applicable, sources of funding for the databases and original study or studies on which the present study is based**	**20**	**Acknowledgements**
**19**	**Data and data sharing**	**Provide the data used to perform all analyses or report where and how the data can be accessed, and reference these sources in the article. Provide the statistical code needed to reproduce the results in the article, or report whether the code is publicly accessible and if so, where**	**19**	**Data Availability statement**
**20**	**Conflicts of Interest**	**All authors should declare all potential conflicts of interest**	**20**	**Competing Interests**

This checklist is copyrighted by the Equator Network under the Creative Commons Attribution 3.0 Unported (CC BY 3.0) license.

Reference

1 Currant, H. *et al.* Genetic variation affects morphological retinal phenotypes extracted from UK Biobank optical coherence tomography images. *PLoS Genet* **17**, e1009497 (2021). https://doi.org/10.1371/journal.pgen.1009497

2 Nalls, M. A. *et al.* Identification of novel risk loci, causal insights, and heritable risk for Parkinson's disease: a meta-analysis of genome-wide association studies. *Lancet Neurol* **18**, 1091-1102 (2019). https://doi.org/10.1016/s1474-4422(19)30320-5

3 Blauwendraat, C. *et al.* Parkinson's disease age at onset genome-wide association study: Defining heritability, genetic loci, and α-synuclein mechanisms. *Mov Disord* **34**, 866-875 (2019). https://doi.org/10.1002/mds.27659

4 Iwaki, H. *et al.* Genomewide association study of Parkinson's disease clinical biomarkers in 12 longitudinal patients' cohorts. *Mov Disord* **34**, 1839-1850 (2019). https://doi.org/10.1002/mds.27845

5 Skrivankova, V. W. *et al.* Strengthening the Reporting of Observational Studies in Epidemiology Using Mendelian Randomization: The STROBE-MR Statement. *Jama* **326**, 1614-1621 (2021). https://doi.org/10.1001/jama.2021.18236

6 Skrivankova, V. W. *et al.* Strengthening the reporting of observational studies in epidemiology using mendelian randomisation (STROBE-MR): explanation and elaboration. *Bmj* **375**, n2233 (2021). https://doi.org/10.1136/bmj.n2233
